# “Step-by-Step” Minimally Invasive Hemostatic Technique Using Intrauterine Double-Balloon Tamponade Combined with Uterine Isthmus Vertical Compression Suture for the Control of Placenta Accreta and Severe Atonic Hemorrhage during a Cesarean Section

**DOI:** 10.1055/s-0041-1733990

**Published:** 2021-08-26

**Authors:** Masaya Takahashi, Jun Takeda, Sumie Haneda, Sumire Ishii, Mitsuko Shinohara, Emiko Yoshida, Anna Sato, Shintaro Makino, Atsuo Itakura

**Affiliations:** 1 Department of Obstetrics and Gynecology, Juntendo University Faculty of Medicine, Tokyo, Japan

**Keywords:** cesarean delivery, atonic hemorrhage, intrauterine double-balloon tamponade, vertical compression suture, minimally invasive hemostatic technique, surgical technique

## Abstract

A sudden onset of postpartum hemorrhage (PPH) during a cesarean delivery requires urgent hemostasis procedures, such as the B-Lynch, Hayman, or double-vertical compression sutures, when bimanual compression, uterotonic agent administration, and intrauterine balloon tamponade had failed to achieve sufficient hemostasis. However, after invasive hemostatic procedures, postoperative complications, including subsequent synechiae and infection followed by ischemia, have been reported to occur even in successful cases. To avoid these complications, we devised and performed a minimally invasive combined technique based on a “step-by-step” minimally invasive hemostatic protocol for a case of placenta accreta and severe atonic hemorrhage during a cesarean delivery. A nullipara woman with a history of systemic lupus erythematosus and treatment with prednisolone and tacrolimus underwent a cesarean section because of a nonreassuring fetal status. Severe atonic hemorrhage and placenta accreta were observed which did not respond to bimanual compression and uterotonics. Because severe uterine atony and continuous bleeding from the placental attachment area were observed even with intrauterine balloon tamponade, vertical compression sutures were placed in the uterine isthmus. However, severe uterine atony and atonic bleeding from the uterine corpus persisted; thus, a second balloon was inserted into the uterine corpus. Hemostasis was accomplished with a combination of isthmus vertical compression sutures and double balloons which is a less-invasive approach than existing compression techniques. No complications related to these procedures were observed. This step-by-step minimally invasive hemostatic technique has the potential to control PPH with less complications, especially in immunocompromised patients.

Critical obstetric hemorrhage remains one of the main causes of maternal death. 1 Thus, rapid and appropriate initial management for controlling massive bleeding is required. As countermeasures against critical obstetric hemorrhage during cesarean deliveries, several hemostatic techniques, such as packing of chitosan covered gauze or iodoform gauze, uterine balloon tamponade (UBT), 2 compression sutures, 3 4 5 arterial ligation, and uterine artery embolization (UAE), 6 have been developed for use in cases of placenta previa or atonic bleeding. Among them, UBT has provided high success rates of hemostasis (∼90%), including in our clinical experiment (93.9%). 7 Clinical practice guidelines worldwide have recommended UBT as the first-line mechanical hemostatic approach for massive intrauterine hemorrhage. 8 9 Various types of compression sutures are available for critical obstetric hemorrhage, including the B-Lynch sutures (conventional compression sutures), 3 Hayman sutures, 4 and double-vertical compression sutures, which we have previously reported. 5 Among these methods, double-vertical compression sutures have been used for critical obstetric hemorrhage during cesarean deliveries in our facility. However, in recent years, in efforts to use less-invasive surgical methods, we have performed isthmus vertical compression suture (IVCS), in which sutures are placed only in the isthmus to avoid excessively reducing blood flow in cases of placenta previa. 10 In addition, we have also reported the efficacy of this minimally invasive method for uterine corpus atonic bleeding. 11 If either UBT or IVCS fails to achieve complete hemostasis, the combination of these techniques is required for further hemostasis. However, we encountered a patient with placenta accreta and severe atonic bleeding who underwent combined UBT and IVCS but failed to achieve hemostasis. Therefore, we devised and performed a novel minimally invasive double-balloon tamponade technique in combination with IVCS without more invasive compression sutures.

## Case Report

**Fig. 1:**
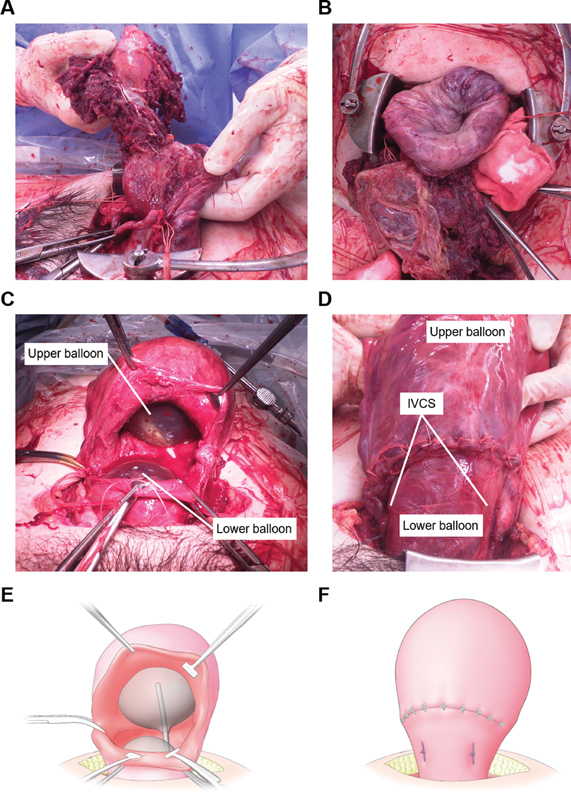
Intraoperative photographs and schematic images during a cesarean section. ( **A, B** ) Uterine inversion due to severe uterine atony and placenta accreta. ( **C, E** ) After IVCS, a lower balloon was inserted through the uterine wound and inflated up to 150 mL. In addition, an upper balloon was inserted into the uterine corpus and inflated up to 500 mL. ( **D, F** ) Postoperative uterine image. The uterine cavity was filled with the two balloons. IVCS, isthmus vertical compression suture.

A 31-year-old nullipara woman with systemic lupus erythematosus (SLE) and antiphospholipid syndrome spontaneously conceived and was referred to our teaching university hospital as a high-risk patient because of oral warfarin use for antiphospholipid syndrome. She was treated with prednisolone 5 mg/day for 11 years and tacrolimus 3 mg/day for 4 years. Warfarin was replaced by subcutaneous self-administration of unfractionated heparin calcium (10,000 IU/day), and low-dose aspirin (100 mg/day) was started from 6 weeks of gestation. At the prenatal checkup, the ultrasonography revealed that there was no placenta previa, and the placenta accreta spectrum was not pointed out. Other than the finding of mild asymmetric fetal growth restriction (about –1.5 standard deviation), her pregnancy course was uneventful. At 37 weeks of gestation, she was admitted to our hospital because of premature rupture of membranes without the onset of labor. Continuous intravenous heparin was started instead of subcutaneous administration to prevent thrombosis which was stopped before labor induction. Labor was induced by intravenous injection of oxytocin. Cesarean delivery was performed because of the lack of change in cervical dilatation of 4 cm for 2 consecutive days. In addition, her body temperature was elevated (>38°C) at the time of surgery, and she was diagnosed with clinical chorioamnionitis. Surgery was performed without any incident until the delivery of the placenta. However, soon after the baby's birth, uterine inversion occurred even without pulling of the umbilical cord, owing to excessive uterine atony. Moreover, as part of the placenta was strongly adhered to the lower posterior wall of the uterus, placenta accreta was diagnosed ( Fig. 1A and B ). Manual removal of the placenta and correction of uterine inversion were successfully performed. As continuous bleeding from the placental attachment area and uterine atony was observed even with the usage of uterotonics, such as oxytocin (10 units) and methylergometrine (0.2 mg), UBT was retrogradely attempted through the opened uterine incision, with the balloon (Fuji Metro; Fuji Latex Co., Ltd. Tokyo, Japan) placed in the uterine isthmus. The balloon was inflated with 300-mL saline, but excessive uterine atony and very thin and edematous myometrium did not allow us to accomplish the hemostasis only with UBT. Therefore, once the balloon was removed, the IVCS was performed. As we previously reported, 10 the two sutures, using 1-Monocryl (Ethicon, Inc., Somerville, NJ), were placed on the uterine isthmus vertically through the anterior to the posterior wall to compress the lower uterine segment. As bleeding continued despite the large decrease in the bleeding amount, the balloon was retrogradely inserted again through the opened uterine incision into the uterine isthmus and inflated with 150-mL saline along with the usage of the uterotonics, and hemostasis was accomplished once. However, because excessive uterine atony and resultant severe atonic bleeding from the uterine corpus occurred again even with bimanual compression, uterotonics, and 1 g of tranexamic acid, a second balloon was retrogradely placed into the uterine corpus through the opened uterine incision and inflated with 500-mL saline to directory press the cavum of the uterine corpus ( Fig. 1C–F ). Complete hemostasis was accomplished using intrauterine double-balloon tamponade in combination with IVCS, and then the uterine incision was closed. A marking stitch was placed on the end of the tube of the lower initial balloon to distinguish the location of the two balloons. The total blood loss was 1,738 mL. The hemoglobin level before the operation was 10.4 g/dL. The lowest hemoglobin level during the procedure was 4.7 g/dL. Transfusion of 8 units of red blood and 6 units of fresh frozen plasma was performed; however, additional compression sutures, UAE and hysterectomy were avoided. With all measures, including blood transfusion, the hemoglobin level after the operation was 8.7 g/dL. For postoperative management, intravenous and oral steroids and continuous intravenous unfractionated heparin (10,000 IU/day) followed by an adjusted dose of warfarin (target prothrombin international normalized ratio of 1.5–2.0, requiring 2.5 mg/day warfarin) were administered. On postoperative day 1, the two balloons were deflated and removed (the lower balloon first followed by the upper balloon), with careful attention to recurrent uterine inversion. Uterine inversion, suture-related pelvic pain, and infection due to uterine ischemia were not observed. The patient was discharged from the hospital 16 days after surgery after controlling the dose of warfarin. She continued outpatient postoperative follow-up, with no signs of subsequent suture-related complications at the 6-month follow-up visit.

## Discussion

This report describes an effective minimally invasive technique combining IVCS and double-balloon tamponade which could accomplish hemostasis in cases of placenta accreta with severe atonic bleeding.

**Fig. 2:**
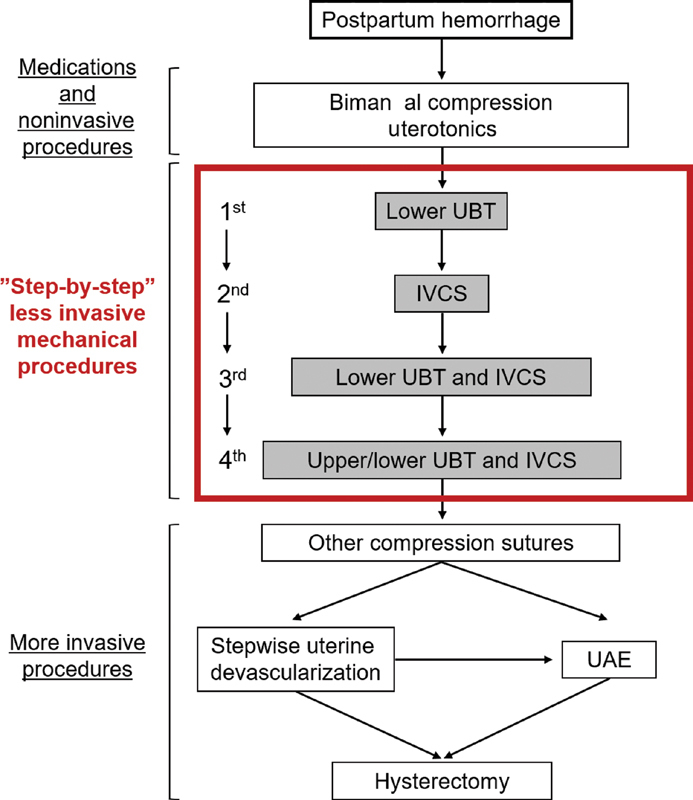
Step-by-step minimally invasive hemostasis protocol. In cases of insufficient hemostasis after medications and noninvasive procedures, step-by-step less-invasive mechanical procedures are prioritized over more invasive procedures. Other compression sutures include the B-Lynch sutures, Hyman sutures, and double-vertical compression sutures. UAE, uterine artery embolization; UBT, uterine balloon tamponade; IVCS, isthmus vertical compression suture.

**Fig. 3:**
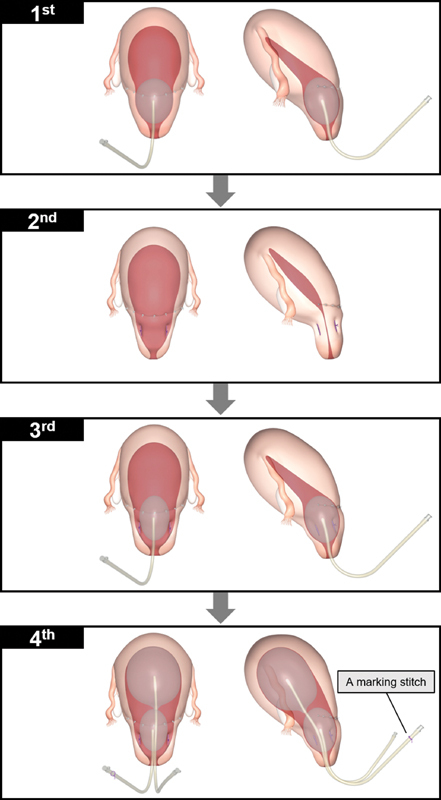
Schematic three-dimensional images of step-by-step minimally invasive hemostasis procedures. Conceptual three-dimensional designs are shown according to our step-by-step minimally invasive hemostasis protocol: front view (left side) and side view (right side). A marking stitch (purple thread) is placed on the end of the tube of the lower initial balloon to distinguish the location of the two balloons.

This technique involves three different mechanisms as follows: (1) Ferguson's reflex (through a lower balloon), (2i) reduction of blood flow (through IVCS), and (3) direct compression of the bleeding area (through an upper balloon). Less-invasive surgical procedures should be performed, especially in patients with high-risk conditions such as autoimmune disorders. Thus, we suggest that this “step-by-step” minimally invasive hemostatic technique ( Figs. 2 and 3 ) may be beneficial, especially as a less-invasive method for controlling postpartum hemorrhage (PPH).

### Step-by-Step Minimally Invasive Hemostasis Protocol

Clinical practice guidelines worldwide have recommended UBT as the first-line method for controlling massive intrauterine hemorrhage because of its high success rates. 8 9 Makino et al. also recommended performing a balloon tamponade test to assess the need for further treatments. 12 Thus, we first performed lower balloon tamponade as the “first mechanical hemostatic step” ( Figs. 2 and 3 ) for placenta accreta and atonic hemorrhage. The mechanism of hemostasis by lower balloon tamponade can be explained by the Ferguson reflex (mechanism 1) which causes uterine contractions by increasing the oxytocin level through the neurohormonal response when lower uterine segment/isthmus or cervical distention occurs. 13 A previous study demonstrated that metreurynters (Fuji Metro) required a median volume of only 120 mL for a balloon tamponade test. 7 In addition, this result was supported by a curvilinear relationship between intraluminal pressure and the volume of Bakri's balloon, the intraluminal pressure of which reached almost a peak at only 50-mL insufflation even if the balloon was inflated up to 500 mL in a previous study. 14 These results explain the effectiveness of lower volume balloon tamponade in the lower uterine segment through the mechanism of the Ferguson reflex.

In our case, as the balloon tamponade test failed to achieve sufficient hemostasis, IVCS was performed as the “second mechanical hemostatic step” ( Figs. 2 and 3 ) to mildly reduce the blood flow to the uterus (mechanism 2). However, in our case, continuous bleeding from the attachment area required the combined use of UBT and IVCS as the “third mechanical hemostatic step” ( Figs. 2 and 3 ) to accomplish hemostasis through both the Ferguson reflex and reduction of blood flow (mechanisms 1 and 2), and hemostasis of the lower segment was successfully achieved. In our facility, these methods (first, second, and third mechanical hemostatic steps) were substantially effective for cases of placenta previa and/or atonic hemorrhage. However, as severe uterine atony with thin myometrium and atonic hemorrhage were observed in the present patient, additional strategies for the next mechanical hemostatic step were needed. As the patient had SLE and was taking steroids and immunosuppressants, she was considered a compromised patient. Therefore, as a less-invasive procedure, a second (upper) balloon was inserted into the uterine corpus and inflated up to 500 mL to directly compress the bleeding area (mechanism 3) as the “fourth mechanical hemostatic step” ( Figs. 2 and 3 ). A recent study demonstrated the effectiveness of direct compression of the intrauterine arterial bleeding area to stop bleeding using two balloons. 15 Complete hemostasis was accomplished using all three mechanisms by following the step-by-step minimally invasive hemostasis protocol. Moreover, no adverse events associated with these procedures were observed. In cases of insufficient hemostasis, even if the above techniques are performed, further invasive methods should be considered ( Fig. 2 ).

### Patients with Systemic Lupus Erythematosus Have Various Perinatal Risks Including Unresponsiveness to Uterotonics Because of a Very Thin Myometrium

SLE is an autoimmune disorder that develops mainly in women of reproductive age and sometimes becomes life-threatening. 16 In addition to the known risks of perinatal events in patients with SLE, some recent reports demonstrated that cases of unresponsiveness to oxytocin 17 18 and excessive uterine atony due to a very thin myometrium 18 19 20 (both present in our patient) were observed in patients with SLE. Moreover, Noh et al reported a case of spontaneous rupture of an unscarred uterus at 23 weeks of gestation in a woman with SLE with long-term steroid treatment. 20 Some studies have explained the possible mechanisms of a thin myometrium. Previous studies demonstrated that 45% of patients with SLE had blocking antibodies to estrogen receptors of the uterine muscle which induced cell-cycle progression and prevented the apoptotic cascade. 21 22 Furthermore, long-term steroid use itself might have an adverse effect of suppressing the estrogen receptor. 23 Tokushige et al reported the pathologic findings of the uterine myometrium after a hysterectomy because of severe uterine atony. They observed uterine fibrosis in a patient with SLE who received long-term steroid treatment, and suggested that uterine fibrosis might have caused PPH in the patient. 18 In our case, the patient had taken prednisolone 5 mg/day for 11 years which was considered long-term steroid treatment.

Although steroid therapy is widely used as a treatment for patients with SLE, there is a concern about infection. 24 Intriguingly, recent studies have revealed that not only the daily dosage but also the total amount of prednisolone pose a high risk of infection. 25 26 As 7% of perinatal deaths were caused by infection in Japan, 27 28 special attention should be paid, especially to pregnant and postpartum patients with autoimmune disorders and steroid use. In our case, no complications associated with infection were observed, although the patient was treated with both long-term steroids and immunosuppressants. Therefore, this minimally invasive procedure may contribute to the safe management of infections. The diagnosis of placenta accreta or PPH before surgery is difficult; thus, counterplans for these unpredictable situations during cesarean deliveries should be considered.

### Our Step-by-Step Minimally Invasive Hemostatic Technique Might Be Able to Effectively and Less Invasively Control Postpartum Hemorrhage during a Cesarean Section

Although several lines of evidence have demonstrated the effectiveness of compression sutures and modified compression sutures, such as the B-Lynch, Hayman, or double-vertical compression sutures, they are associated with some complications, including infection and uterine necrosis. 29 30 Surprisingly, a previous report described the case of a patient in whom the B-Lynch sutures did not improve uterine atonic hemorrhage because of excessive atony and a very thin myometrium. 18 In addition, although UAE, which is less invasive than hysterectomy, has been reported to be highly effective, it has been associated with risks of severe complications, including uterine necrosis and uterine rupture. 31 32 Therefore, our less-invasive hemostatic procedures might reduce these severe complications. In our case, the step-by-step minimally invasive hemostatic technique was efficiently accomplished using the combination of minimally invasive techniques with different mechanisms.

## Conclusion

Although additional cases are needed to clarify its effectiveness, this step-by-step minimally invasive hemostatic technique has the potential to less invasively control severe PPH during cesarean deliveries.
